# One-stage total aortic arch replacement with four-branched frozen elephant trunk graft and thoracic endovascular aortic repair using the telescope technique

**DOI:** 10.1016/j.xjtc.2026.102379

**Published:** 2026-03-27

**Authors:** Hiroto Yasumura, Koichiro Shimoishi, Yoshihiro Fukumoto, Goichi Yotsumoto, Yuki Ogata, Tomoyuki Matsuba, Yoshiharu Soga

**Affiliations:** a Department of Cardiovascular Surgery, Kagoshima City Hospital, Kagoshima, Japan; b Department of Cardiovascular Surgery, Graduate School of Medical and Dental Sciences, Kagoshima University, Kagoshima, Japan

**Figure:**
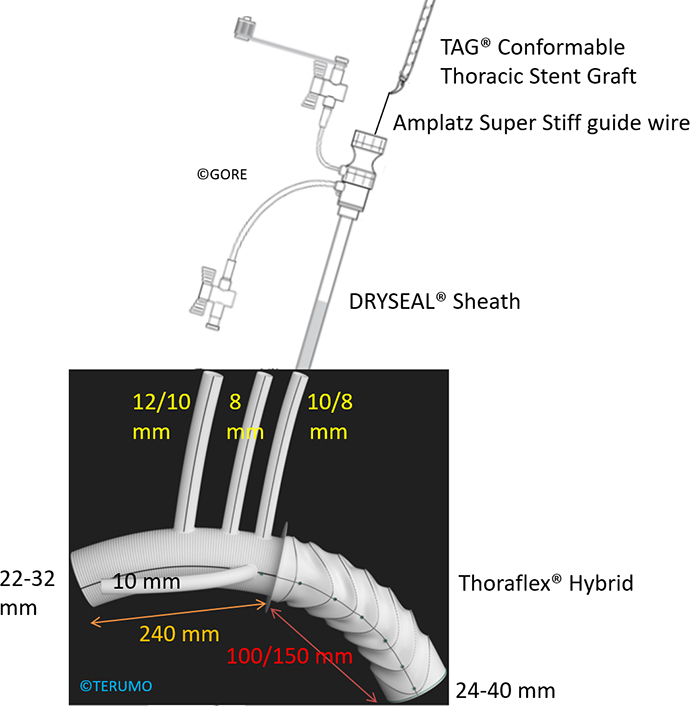
Image of one-stage TAR with a 4-branched FET graft and TEVAR using the telescope technique.

**Central Message**

One-stage TAR with a 4-branched FET graft and TEVAR using the telescope technique is applicable for aneurysms from the aortic arch to the descending aorta, in particular with poor vascular access.

The Thoraflex Hybrid (Terumo Aortic) is a novel 4-branched frozen elephant trunk (FET) graft that eliminates the need for suturing between the branched graft and an open stent graft, enabling a circulatory arrest time within 30 minutes.^1^ In patients with aneurysms extending from the aortic arch to the descending aorta, total aortic arch replacement (TAR) with FET technique, and 2-staged thoracic endovascular aortic repair (TEVAR) may be effective.^2^ However, 1-stage TAR with a 4-branched FET graft and TEVAR using the telescope technique (Figure 1) is a more universal and time-saving strategy, in particular with poor femoral access. Our patient provided consent for the publication of this article.

**Figure 1:**
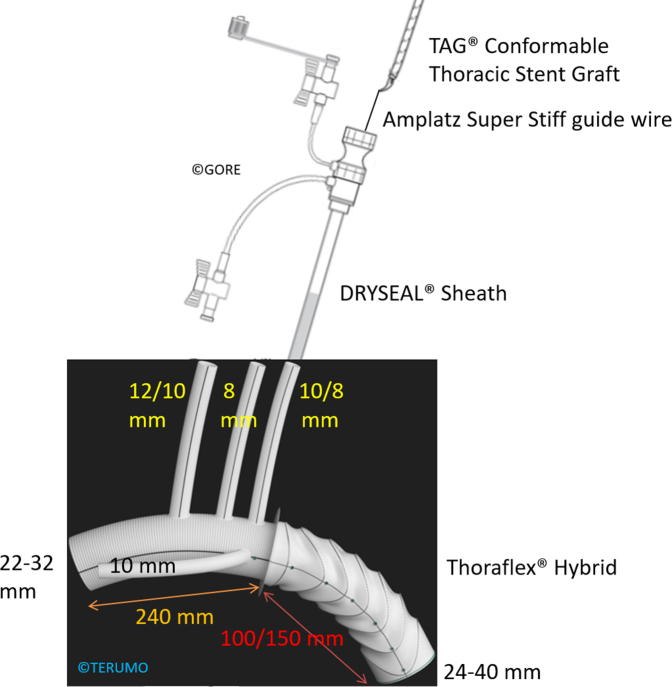
Image of 1-stage TAR with a 4-branched FET graft (Thoraflex Hybrid prosthesis) and TEVAR using the telescope technique. Each Thoraflex Hybrid prosthesis has a 10-mm neck branch, through which a DRYSEAL Flex Introducer Sheath of 24F or less (outer diameter: 8.8 mm) can be inserted.

## Surgical Technique

**Figure 2:**
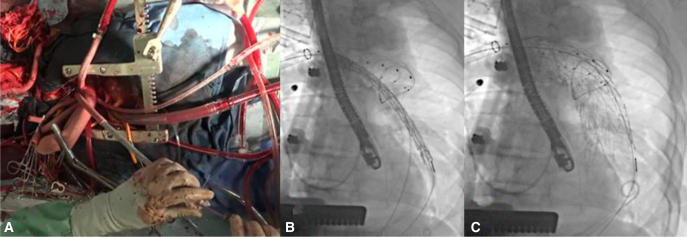
Intraoperative findings: A, A 24F (outer diameter: 8.8 mm) DRYSEAL Flex Introducer Sheath (WL Gore & Associates) was inserted through a 10-mm branch of the Thoraflex Hybrid prosthesis (TX-P3038100;Terumo aortic). B, A GORE TAG Conformable Thoracic Stent Graft (TGM454515J; WL Gore & Associates) was advanced over an Amplatz Super Stiff guidewire (Boston Scientific) and telescoped into the stent graft portion of the Thoraflex Hybrid prosthesis. C, The GORE TAG Conformable Thoracic Stent Graft was deployed above the aortic valve level.

Under general anesthesia, median sternotomy is performed and cardiopulmonary bypass (CPB) is established. Core cooling is started, and a vent cannula is placed in the left ventricle (Video 1). After complete circulatory arrest, incision of the ascending aorta and transection of the aortic arch at zone 1 or 2 is performed with antegrade or retrograde cerebral perfusion and coronary perfusion. Then, the stent graft component of the Thoraflex Hybrid prosthesis is deployed in the descending aortic aneurysm. The collar of the Thoraflex Hybrid prosthesis is anastomosed to the transected aortic arch with an open distal anastomosis technique. Systemic circulation is resumed via a branch of the Thoraflex Hybrid prosthesis. A DRYSEAL Flex Introducer Sheath (WL Gore & Associates) is inserted through another branch of the Thoraflex Hybrid prosthesis under fluoroscopic guidance (Figure 2, *A*). The thoracic stent graft is advanced over a stiff guidewire, telescoped into the stent graft portion of the Thoraflex Hybrid prosthesis (Figure 2, *B*), and deployed in the descending aorta (Figure 2, *C*). Reconstruction of the neck branches and proximal anastomosis of the Thoraflex Hybrid prosthesis are then performed, followed by weaning from CPB.

## Discussion

**Figure 3:**
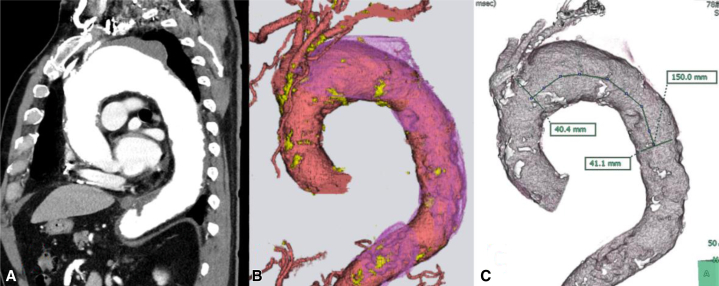
Preoperative findings of Figure 2. A, Contrast-enhanced computed tomography (*CT*) revealed a saccular distal aortic arch aneurysm measuring 64 mm in diameter and a fusiform descending aortic dilatation measuring 42.6 mm in diameter. B, Three-dimensional CT of the aneurysm. C, Measurement of the aortic diameter and length on 3-dimensional CT. Even with the use of a 150-mm stent graft of the Thoraflex Hybrid prosthesis, the descending aortic landing zone measured 41.1 mm in diameter, exceeding the applicable size for the device.

In our hospital, the 4-branched FET graft is routinely used for distal aortic arch aneurysms, enabling distal anastomosis under circulatory arrest within 30 minutes, as previously reported. Figure 3, *A* and *B*, shows a case of distal aortic arch aneurysm and dilation of the proximal descending aorta, and 1-stage TAR with a Thoraflex Hybrid prosthesis and TEVAR using the telescope technique was performed (Figure 2). Narrow, tortuous, and heavily calcified access routes can cause complications such as dissection, occlusion, or rupture.^3^ The access vessels in the current case were calcified and narrow, and we predicted that TEVAR via the femoral and iliac arteries was challenging. Therefore, the telescope technique, with which the access route is not restricted from device size, was reasonable and applicable. TEVAR via a branch of the Thoraflex Hybrid prosthesis using the telescope technique is not limited by the diameter of introducer sheaths and thoracic stent grafts. The Thoraflex Hybrid prosthesis has branches, measuring 8 mm, 10 mm, and 12 mm in diameter (Figure 1), which encompass all sizes of the DRYSEAL Sheath.

Paraplegia, a serious complication of circulatory arrest, is associated with prolonged circulatory arrest time^4^; thus, the Thoraflex Hybrid prosthesis may reduce this risk. The saved time can be used for additional TEVAR in the descending aorta. However, the increased coverage of the descending aorta afforded by the telescope technique may conversely increase the risk of paraplegia; therefore, preoperative identification of the Adamkiewicz artery is imperative.

Furthermore, TEVAR via a branch of the Thoraflex Hybrid prosthesis is not limited by the diameter or length of the landing descending aorta. The maximum stent graft size of the Thoraflex Hybrid prosthesis is 40 mm in diameter and 150 mm in length; however, the 40-mm-diameter stent graft is made to order and not always available.^5^ In the current case, the 40-mm-diameter stent graft of the Thoraflex Hybrid prosthesis was unavailable, and even the 150-mm-long stent graft was insufficient because its descending aortic landing zone measured 41.1 mm in diameter (Figure 3, *C*), exceeding the applicable range. Therefore, we used a 38-mm-diameter, 100-mm-long stent graft of the Thoraflex Hybrid prosthesis as a scaffold for the subsequent thoracic stent graft. Because the branches of the Thoraflex Hybrid prosthesis cover all sizes of DRYSEAL Sheath and thoracic stent graft, the appropriate size of the thoracic stent graft can be selected depending on the landing descending aorta even under a poor access route.

## Conclusions

One-stage TAR with a four-branched FET graft and TEVAR using the telescope technique is applicable in all patients with aneurysms from the aortic arch to the descending aorta. It is feasible in cases with challenging vascular access for staged TEVAR and in cases where the maximum stent graft diameter of the branched FET is undersized for the descending aorta.

## Conflict of Interest Statement

The authors reported no conflicts of interest.

The *Journal* policy requires editors and reviewers to disclose conflicts of interest and to decline handling or reviewing manuscripts for which they may have a conflict of interest. The editors and reviewers of this article have no conflicts of interest.

## Supplementary Data

Video 1 Preoperative CT images and intraoperative findings. Video available at: https://www.jtcvs.org/article/S2666-2507(26)00186-0/fulltext.
