# Supplementary figures and images for: One-stage total aortic arch replacement with four-branched frozen elephant trunk graft and thoracic endovascular aortic repair using the telescope technique
Source: JTCVS Tech. 2026 Mar 27;37:102379. doi: 10.1016/j.xjtc.2026.102379 (PMC13261201; doi:10.1016/j.xjtc.2026.102379)

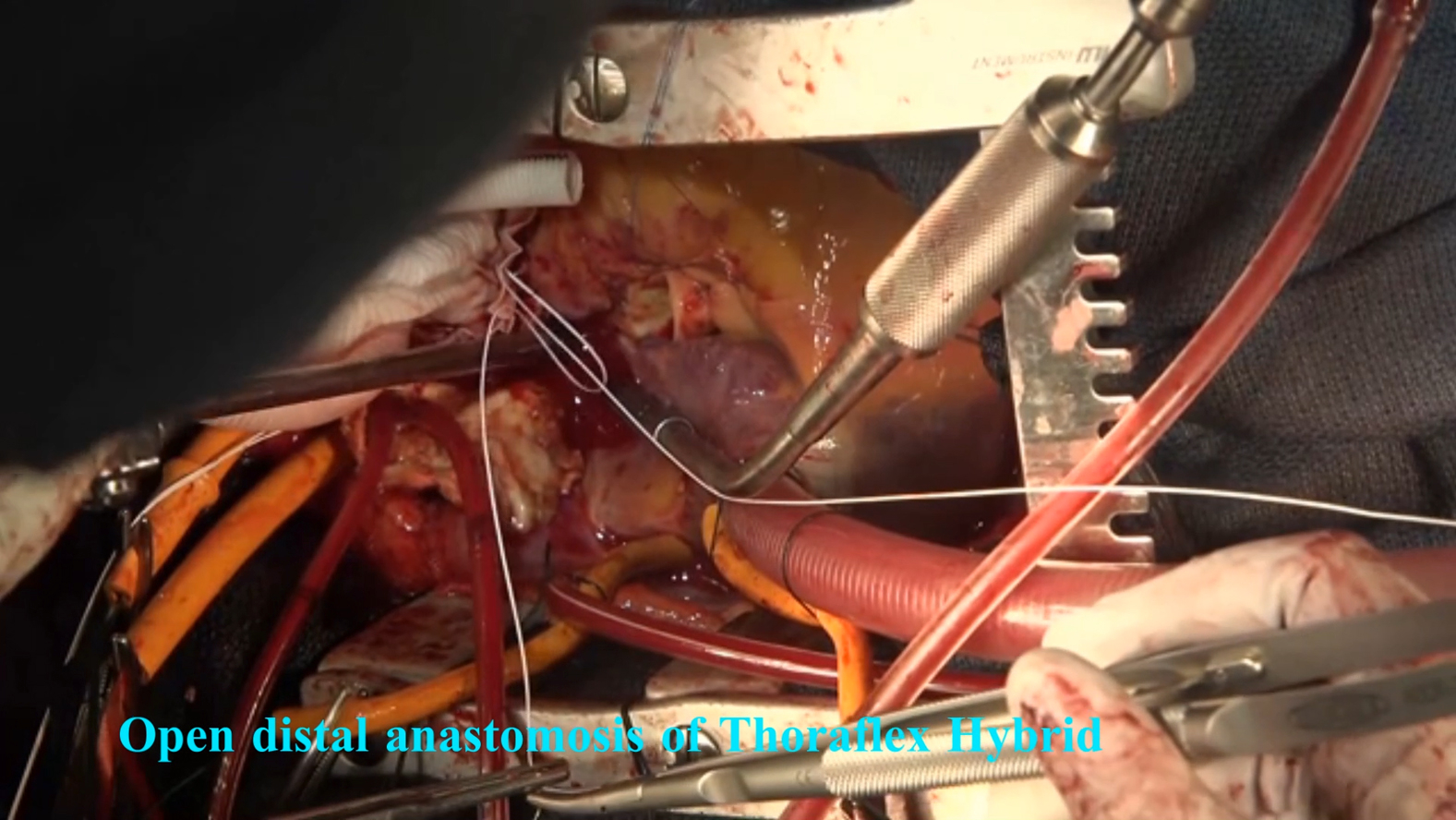

Supplement: Video 1 — Preoperative CT images and intraoperative findings. Video available at: https://www.jtcvs.org/article/S2666-2507(26)00186-0/fulltext. [file fx2.jpg]
